# Supplementary material for: Genetic requirement for Esrp1 and Esrp2 in vertebrate pituitary morphogenesis
Source: Development. 2025 Oct 30;152(21):dev204636. doi: 10.1242/dev.204636 (PMC12633794; doi:10.1242/dev.204636)
Supplement: Supplementary information [file develop-152-204636-s1.pdf]

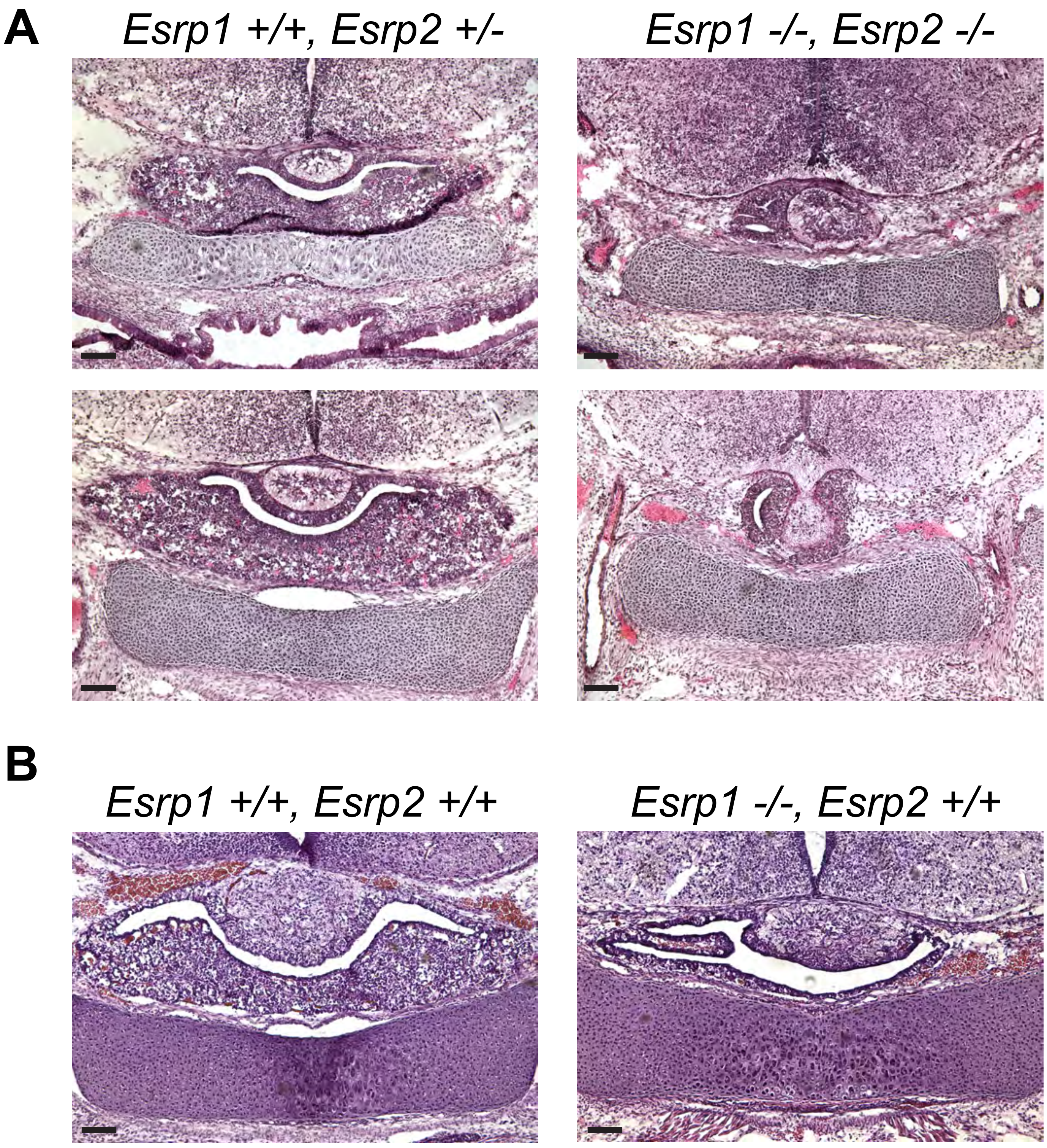

Fig. S1. ***Esrp1-/-,Esrp2-/-* and *Esrp1-/-,Esrp2+/+* mice have an absent or severely hypoplastic anterior pituitary. A**) Coronal H&E stained sections of E17.5 littermate control (*Esrp1* +/+, *Esrp2* +/-) and *Esrp1-/-, Esrp2-/-* null pituitary. Two control and 2 null embryos are shown. N=2. **B**) Coronal H&E stained sections of E17.5 littermate control (*Esrp1* +/+, *Esrp2* +/+) and *Esrp1-/-,Esrp2+/+* pituitary. N=1. Scale bar = 100 μm.

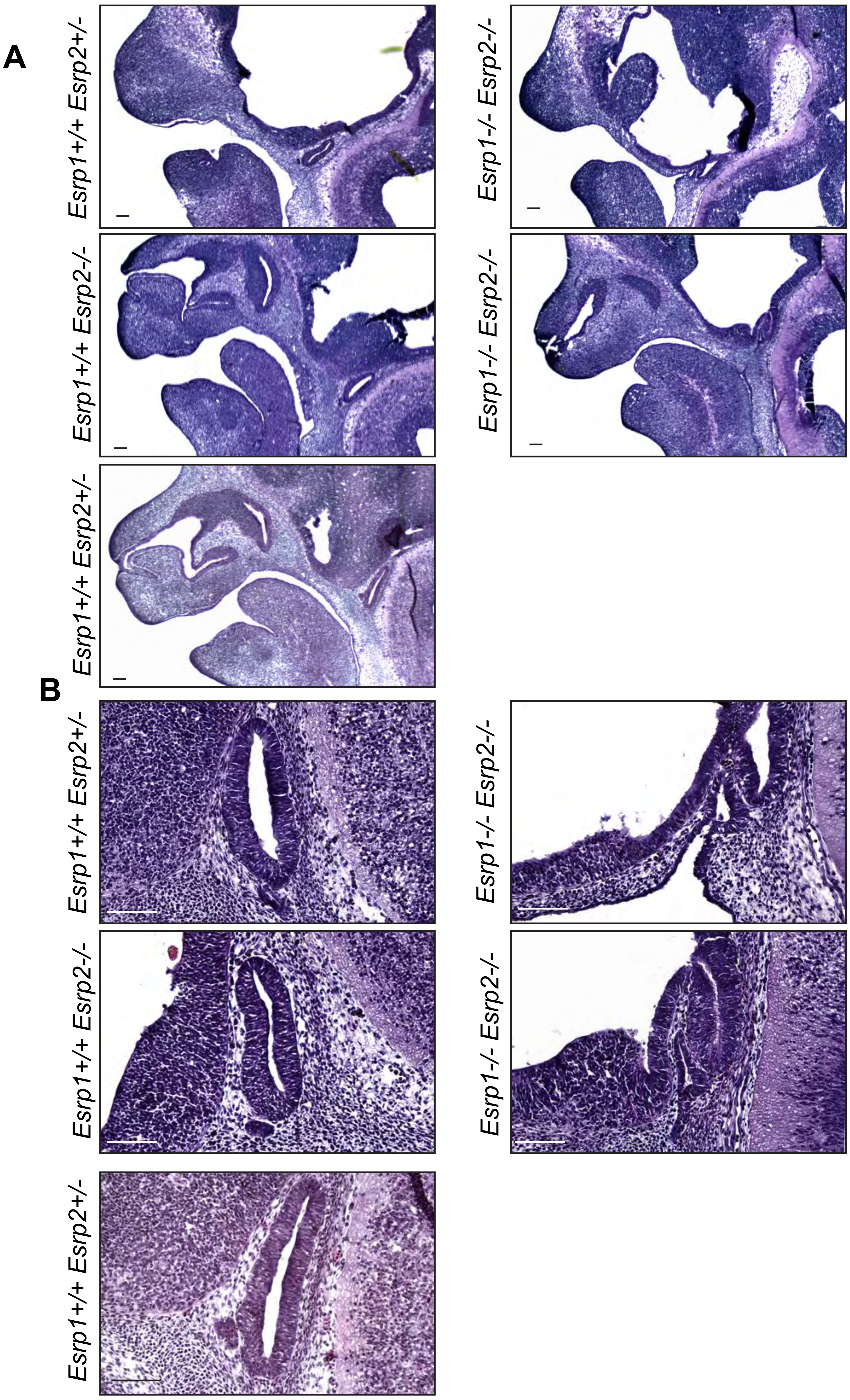

Fig. S2. *Esrp1-/-,Esrp2-/-* null mice have impaired Rathke's pouch formation.

Sagittal sections of Rathke's pouch in three E12.5 littermate controls and two *Esrp1-/-, Esrp2-/-* null mouse embryos. **A**) H&E staining shows Rathke's pouch of *Esrp1-/-, Esrp2-/-* null embryos to be significantly smaller and dysmorphic relative to controls. Scale bar = 0.1 mm. **B**) Increased magnification of Rathke's pouch. Scale bar = 100 μm.

Development • Supplementary information

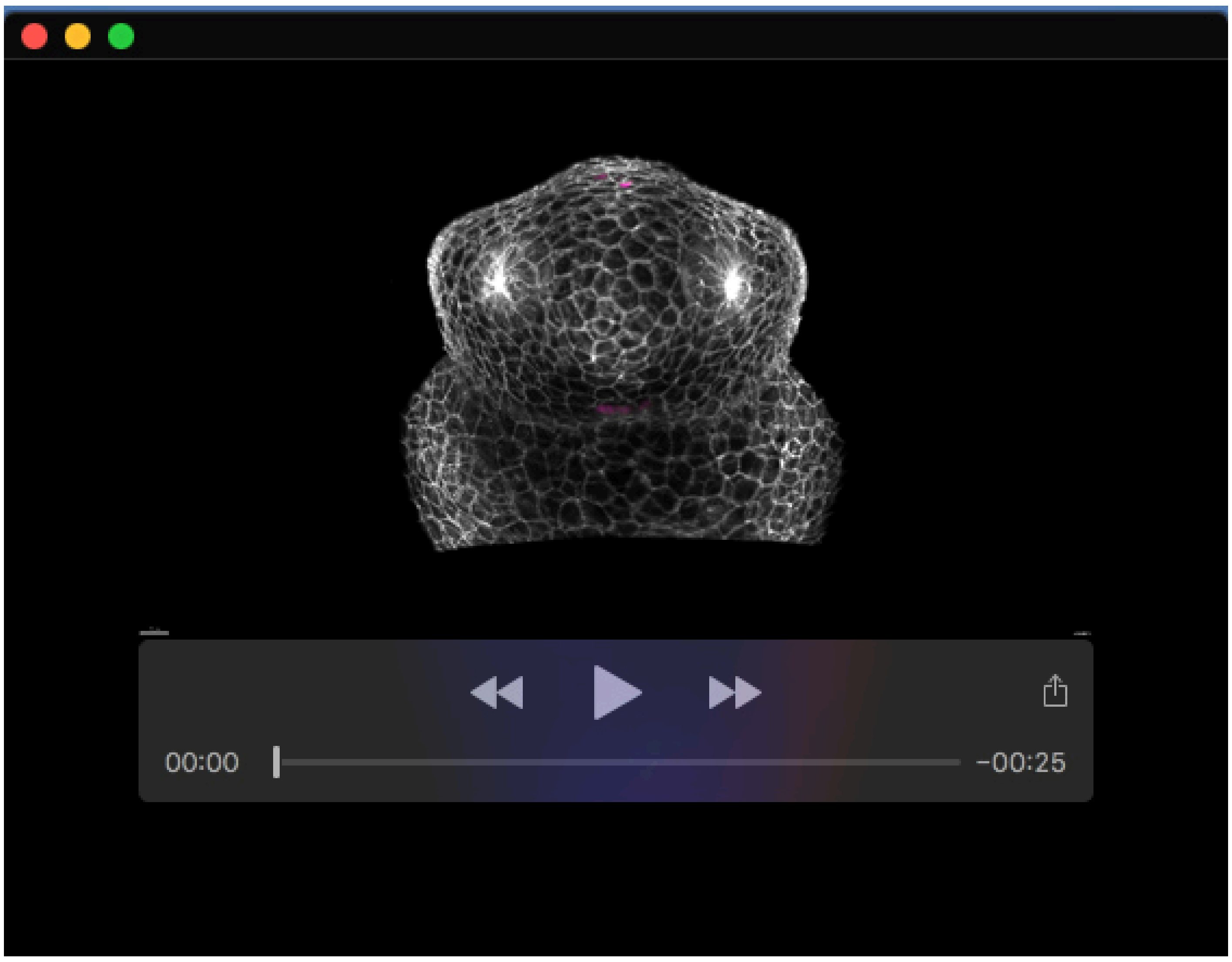

Movie 1. Frontal view light-sheet microscopy (LSM) imaging of 27 hpf zebrafish expressing *lhx3*:tdTomato (magenta) and *cdh1*:gfp (gray). Images were collected every 15 minutes for 15 hours.

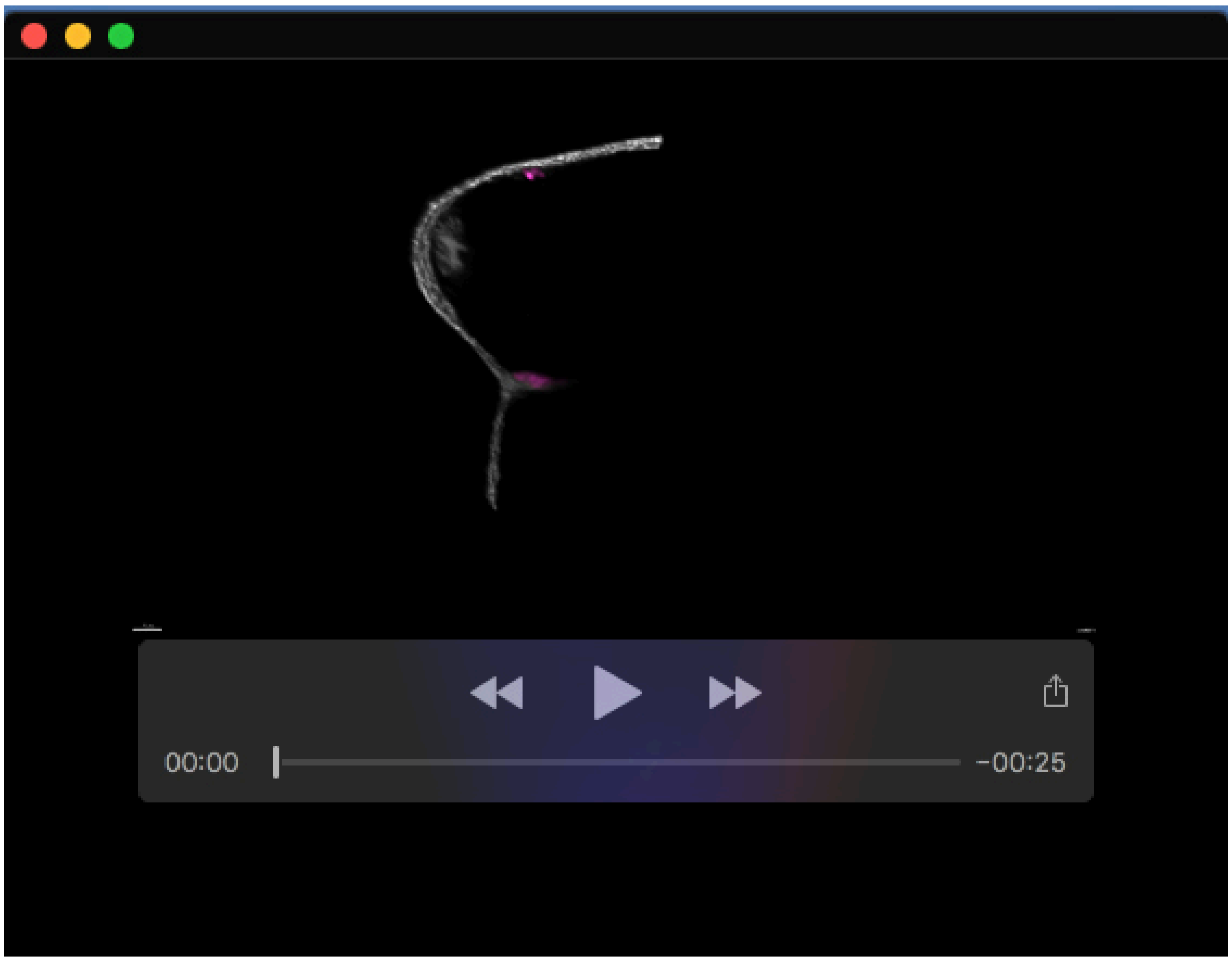

Movie 2. Medial sagittal optical section of lateral view light-sheet microscopy (LSM) imaging of 27 hpf zebrafish expressing *lhx3*:tdTomato (magenta) and *cdh1*:gfp (gray). Images were collected every 15 minutes for 15 hours.

Development • Supplementary information

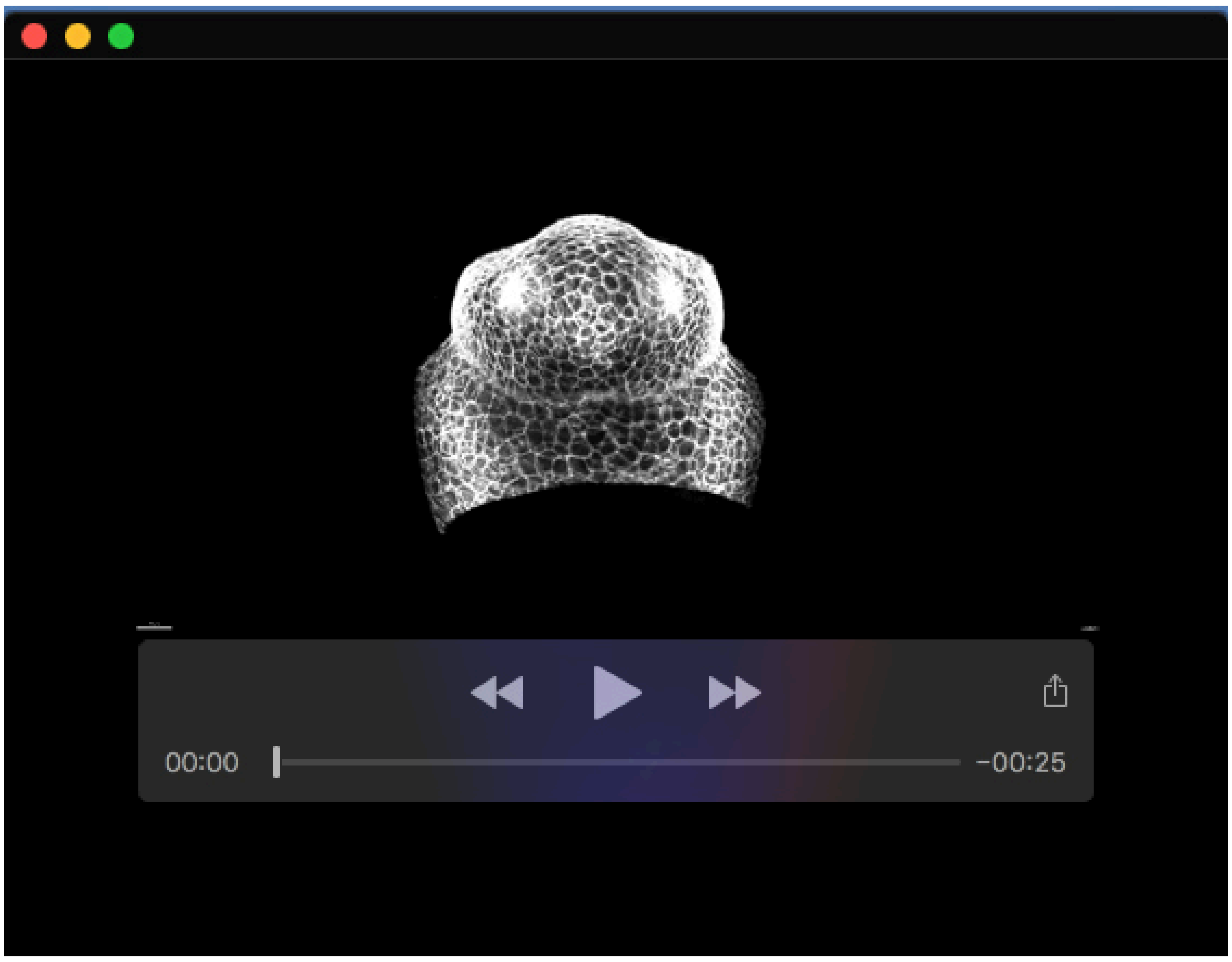

Movie 3. Frontal view light-sheet microscopy (LSM) imaging of 27 hpf zebrafish from Movie 1 with only *cdh1*:gfp (gray) expression displayed.

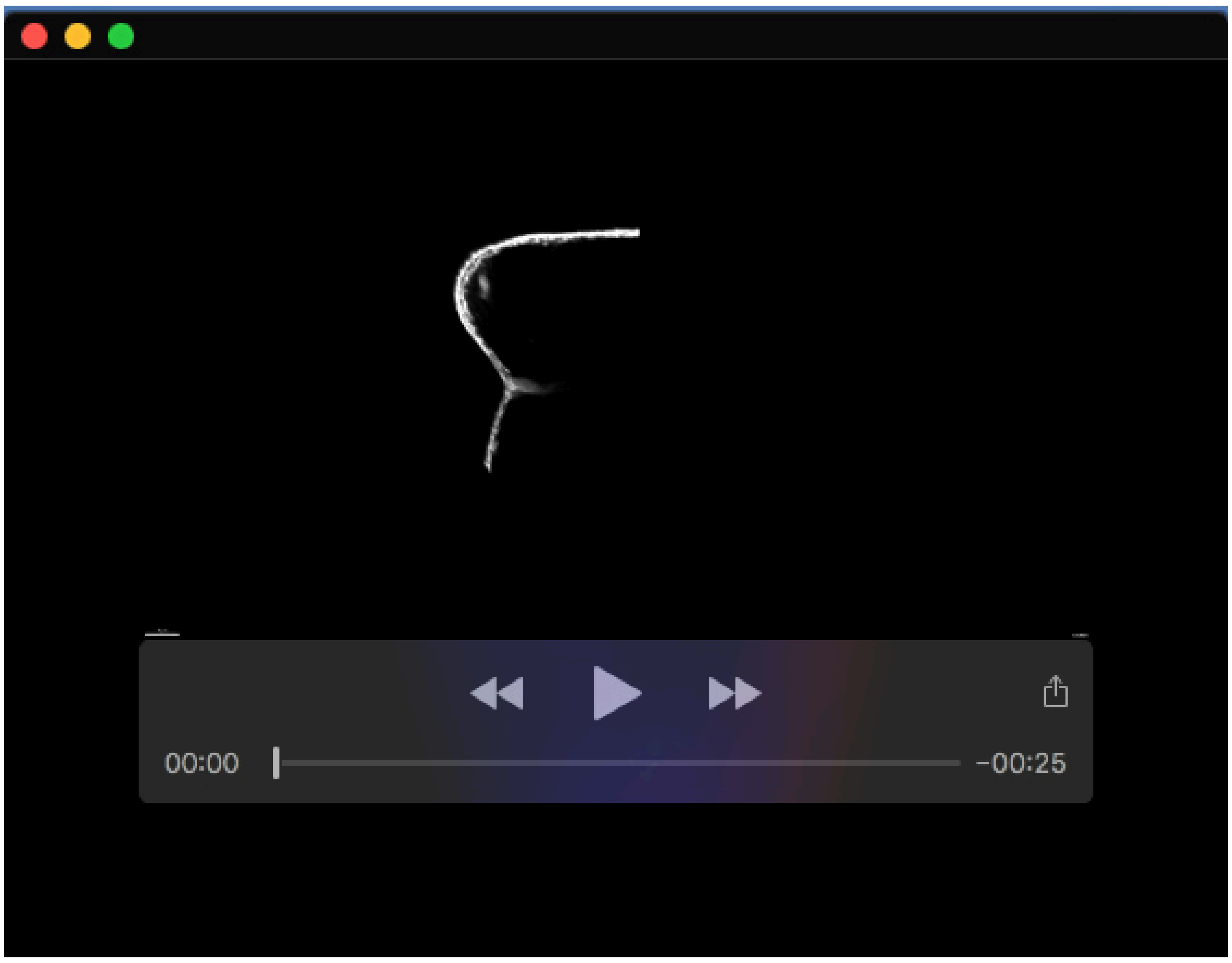

Movie 4. Medial sagittal optical section of lateral view light-sheet microscopy (LSM) imaging of 27 hpf zebrafish from Movie 2 with only *cdh1*:gfp (gray) expression displayed.

Development • Supplementary information
